# Impact of Thyroid Autoimmunity on *In Vitro* Fertilization/Intracytoplasmic Sperm Injection Outcomes and Fetal Weight

**DOI:** 10.3389/fendo.2021.698579

**Published:** 2021-07-08

**Authors:** Ning Huang, Lixue Chen, Ying Lian, Haining Wang, Rong Li, Jie Qiao, Hongbin Chi

**Affiliations:** ^1^ Center for Reproductive Medicine, Department of Obstetrics and Gynecology, Peking University Third Hospital, Beijing, China; ^2^ Department of Endocrinology and Metabolism, Peking University Third Hospital, Beijing, China

**Keywords:** thyroid autoimmunity, *in vitro* fertilization/intracytoplasmic sperm injection, thyroid antibodies, pregnancy outcomes, fetal weight

## Abstract

Several studies have reported the association between thyroid autoimmunity (TAI) and *in vitro* fertilization (IVF)/intracytoplasmic sperm injection (ICSI) outcomes. However, the findings remain controversial. We performed a large-scale retrospective cohort study to verify the effect of the presence of thyroid antibodies on IVF/ICSI outcomes and fetal growth and to evaluate the association between the types and titers of thyroid antibodies and adverse IVF/ICSI outcomes. A total of 16481 patients with infertility were referred to the Reproductive Center of Peking University Third Hospital for their first IVF/ICSI treatment between January 2018 and June 2019.Patients who sought IVF/ICSI treatment due to tubal or male factors infertility and who achieved fresh embryo transfer were included in our study. Finally, 778 patients with thyroid antibody positivity were selected as the TAI group, and 778 age-matched patients were included in the control group. The number of oocytes retrieved and high-graded embryos and the rates of clinical pregnancy, miscarriage, live birth, and preterm delivery were compared between the TAI and control groups. In addition, subgroup analysis was performed to demonstrate whether different types and titers of thyroid antibodies had different effects on IVF/ICSI outcomes. After adjusting for thyroid function, anti-Müllerian hormone levels, basal follicle stimulating hormone levels, basal estradiol levels and antral follicle count, the number of oocytes retrieved in the TAI group was significantly lower than that in the control group. No significant differences were observed between the two groups in the rates of clinical pregnancy, miscarriage, preterm delivery, live birth, and birth weight in singletons; however, the birth weight in twin pregnancy was significantly lower in the TAI group than in the control group. Subgroup analysis showed no association between the types or titers of thyroid antibodies and adverse IVF/ICSI outcomes. In conclusion, the presence of TAI in patients with infertility did not impair embryo quality or affect pregnancy outcomes, including clinical pregnancy, miscarriage, preterm delivery, and live birth. However, it decreased the number of oocytes retrieved and birth weight in twin pregnancy.

## Introduction

Thyroid autoimmunity (TAI), diagnosed as the presence of thyroid antibody, is the most common autoimmune disorder among women of childbearing age. Accumulating evidence has demonstrated the association between thyroid antibody and pregnancy outcomes after *in vitro* fertilization (IVF)/intracytoplasmic sperm injection (ICSI) treatment, and studies based on the IVF/ICSI process have not only provided new models to investigate the association between thyroid antibodies and pregnancy outcomes but also enabled the analysis of the role of TAI in earlier gestational stages from oocyte fertilization to embryo implantation (1). For example, some studies showed that euthyroid women with TAI had a higher risk of adverse outcomes, such as miscarriage and preterm delivery, than healthy women without TAI, but other studies did not support the association. In addition, in a meta-analysis, including 12 studies, Andrea et al. performed a comprehensive analysis on the association between TAI and IVF/ICSI outcomes (2), and they concluded that the presence of thyroid antibodies exerted detrimental effects on pregnancy outcomes, including an increased risk of miscarriage and a decreased frequency of live birth. However, all the aforementioned studies failed to show any association between the presence of thyroid antibodies and the number of oocytes retrieved, fertilization rate, or implantation rate. Moreover, the sample sizes in the studies included in this meta-analysis were all small, and the conclusions in those studies were controversial.

Thyroglobulin and thyroid peroxidase function as key factors in the process of thyroid hormone (TH) biosynthesis and constitute the major thyroid autoantigens involved in the pathophysiology of TAI (3). The prevalence of thyroid peroxidase antibody (TPOAb) and thyroglobulin antibody (TGAb) in patients with TAI is similar; however, the sensitivity and titers of TPOAb are always higher than those of TGAb (4). A large cross-sectional study reported that women with high titers of TPOAb or TGAb displayed impaired capacity of the thyroid gland adapting to enlarged demands during pregnancy, and the thyroid function was more prone to be affected in women with co-positive antibodies of TPOAb and TGAb than in those with isolated-positive antibody (5).

The present large retrospective cohort study aimed to investigate whether the presence of thyroid antibody is associated with IVF/ICSI outcomes, including the number of oocytes retrieved, embryo quality, clinical pregnancy rate, miscarriage rate, preterm delivery rate, live birth rate, and birth weight. In addition, subgroup analysis was performed to determine the association between the types and titers of thyroid antibody and pregnancy outcomes after IVF/ICSI.

## Methods

### Study Population

**Figure 1:**
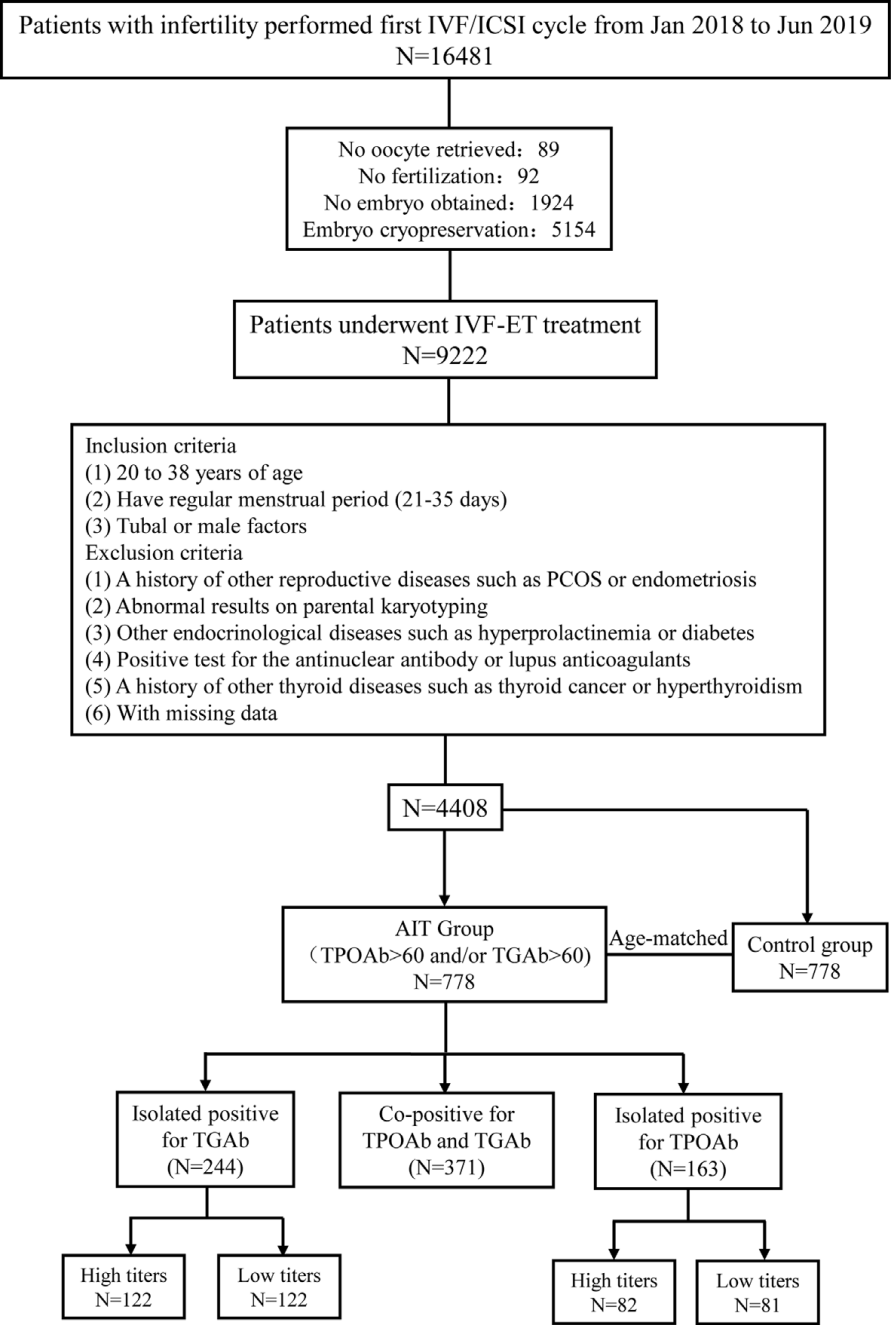
Flow chart of study cohort selection. IVF/ICSI, *in vitro* fertilization/intracytoplasmic sperm injection; TGAb, thyroglobulin antibody; TPOAb, thyroid peroxidase antibody.

A total of 16481 patients with infertility were referred to the Reproductive Center of Peking University Third Hospital for their first IVF/ICSI treatment between January 2018 and June 2019. After excluding 89 patients with no oocyte retrieved, 92 patients without fertilization, 1924 patients with no embryo obtained, and 5154 patients with embryo cryopreservation, a total of 9222 patients who underwent standardized, controlled ovarian stimulation and achieved fresh embryo transfer were screened for eligibility. Patients were included in our study if they were 20 to 38 years old, had a regular menstrual period (21–35 days), were referred to the reproductive center for the first IVF/ICSI cycle due to tubal or male factors, and underwent fresh embryo transfer. Patients were excluded from the study if they had a history of other reproductive diseases, such as polycystic ovarian syndrome and endometriosis; a history of other thyroid diseases, such as hyperthyroidism or thyroid cancer; abnormal results on parental karyotyping; other endocrinological diseases, such as hyperprolactinemia and diabetes; or positive tests for the antinuclear antibody or lupus anticoagulants. Patients were also ineligible if they had abnormal thyroid function testing, except for thyroid antibody positivity, before IVF/ICSI treatment. A total of 778 patients with thyroid antibody positivity were selected as the TAI group. Women without thyroid antibody positivity were individually matched to a single patient with TAI at a ratio of 1:1 for age and 778 patients were selected in the control group for analysis (**Figure 1**).

### Assisted Reproductive Technology Procedure

All patients received a standardized, controlled ovarian stimulation (COS) regimen, oocyte retrieval, and fertilization, followed by fresh embryo transfer. Patients treated with ultralong-term and long-term protocols received a long-acting gonadotropin-releasing hormone (GnRH) agonist for downregulation. After downregulation was achieved, recombinant gonadotropins were administered for ovarian stimulation. Patients treated with the short-term protocol were simultaneously administered a short-acting GnRH agonist and recombinant gonadotropins for ovarian stimulation. In patients treated with the antagonist protocol, recombinant gonadotropins were initiated on day 2 of the menstrual cycle, and treatment with a GnRH antagonist was initiated when at least one follicle reached 12 mm in diameter and continued until the day when human chorionic gonadotropin (HCG) was administered. The individualized dose of gonadotropins was decided based on the patient’s age, body mass index (BMI), and anti-Müllerian hormone (AMH) levels. Recombinant HCG 250μg (Eiser, Serono, Germany)was administered to trigger oocyte maturation when at least two follicles reached 18 mm. Oocyte retrieval was performed 34 to 36 hours after HCG administration. Insemination was performed at 4 to 6 hours after oocyte retrieval by a conventional *in vitro* fertilization method or intracytoplasmic sperm injection according to the sperm quality. Up to two day-3 embryos or blastocysts were transferred 3 or 5 days after oocyte retrieval.

### Study Outcomes

Clinical pregnancy was defined as at least one gestational sac in the uterus at 35 days after embryo transfer, as observed by ultrasonography. Miscarriage was defined as the loss of clinical pregnancy before 28 weeks of gestation. Live birth was defined as the delivery of at least one survived newborn, irrespective of gestation duration. Preterm delivery was defined as the delivery of a living fetus before 37 weeks of gestation.

### Laboratory Testing

Blood samples for TH testing were taken within 6 months prior to the initiation of COS. Serum thyroid-stimulating hormone (TSH), free thyroxin (FT4), TPOAb, and TGAb levels were measured by a fully automatic chemiluminescence immunoassay analyzer (ADVIA Centaur XP, Siemens Healthcare Diagnostics). The reference values were 0.55–4.78 uIU/ml for TSH and 0.89–1.80 ng/dl for FT4. The level below 60 IU/ml was defined as negative for TPOAb or TGAb.

### Categorization

Based on the different types of antibody positivity, patients in the TAI group were divided into three subgroups: group 1, co-positive for TPOAb and TGAb (TPOAb+ and TGAb+); group 2, isolated TGAb-positive (TPOAb- and TGAb+); group 3, isolated TPOAb-positive (TPOAb+ and TGAb-). Then, according to different antibody titers, patients with isolated TGAb positive or isolated TPOAb positive were further divided into the high and low titers groups based on sample size (**Figure 1**).

### Statistics

Continuous data are shown as mean (standard deviation [SD]) for normally distributed data and median (interquartile range) for non-normally distributed data. Categorical data are presented as the number of cases (percentage). Continuous variables were compared using the Student’s t-test or one-way ANOVA, and categorical variables were compared using the chi-square test. Continuous variables without normal distributions were compared using the Mann-Whitney U test. Logistic regression analysis was conducted to calculate odds ratios (ORs) with 95% confidence intervals (CIs) after adjusting for relevant factors. Linear regression model was performed to analyze the association between the number of oocytes retrieved and relevant factors. All analyses were performed using SPSS 24 statistical software. Statistical significance was defined as two-sided P value < 0.05.

## Results

As shown in **Table 1**, there was no significant difference between the two groups in BMI or duration of infertility; however, significantly higher AMH levels (median [interquartile range]: 2.5 [1.4–4.2] *vs.* 2.4 [1.4–3.7], P = 0.024) and basal estradiol (E2) levels (median [interquartile range]: 165.0 [131.0–204.0] *vs.* 157.0 [124.3–198.0], P = 0.025) were observed in the TAI group than in the control group, indicating a different ovarian reserve between the two groups. The levels of basal follicle stimulating hormone (FSH) were lower in patients with TAI than those without TAI but the difference was not significant. No significant difference was observed in antral follicle count between two groups. A significant difference was observed in thyroid function between the TAI and control group. The levels of FT4 were significantly lower (mean [SD]: 1.3 [0.2] *vs.* 1.3 [0.1], P = 0.012), but the levels of TSH were significantly higher (median [interquartile range]: 2.1 [1.5–2.9] *vs.* 2.0 [1.4–2.6], P = 0.007) in patients with TAI than in controls.

**Table 1:** Baseline characteristics of patients.

Characteristics	TAI group (N=778)	Control group^a^ (N=778)	P values
Body mass index, mean (SD), kg/m^2^	22.6 (3.4)	22.7 (3.4)	0.386
Duration of infertility, median (IQR), y	3 (2-4.3)	3 (2-4)	0.925
Type of infertility, No. (%)			
Primary	476 (61.2)	429 (55.1)	0.016
Secondary	302 (38.8)	349 (44.9)	
Basal FSH, median (IQR), mIU/ml^b^	6.7 (5.4-8.1)	6.8 (5.7-8.2)	0.063
Basal LH, median (IQR), mIU/ml	3.6 (2.5-4.8)	3.6 (2.5-4.8)	0.555
Basal E_2_, median (IQR), pmol/L	165.0 (131.0-204.0)	157.0 (124.3-198.0)	0.025
AMH, median (IQR), ng/ml	2.5 (1.4-4.2)	2.4 (1.4-3.7)	0.024
Antral follicle count in both ovaries, median (IQR)	9 (7-13)	10 (7-13)	0.465
FT4, mean (SD), ng/dl	1.3 (0.2)	1.3 (0.1)	0.012
TSH, median (IQR), mIU/L	2.1 (1.5-2.9)	2.0 (1.4-2.6)	0.007

FSH, follicle stimulating hormone; LH, luteinizing hormone; E_2_, estradiol; AMH, Anti-Mullerian hormone; FT4, free thyroxine; IQR, interquartile range; SD, standard deviation; TAI, thyroid autoimmunity; TSH, thyroid-stimulating hormone.

^a^The control and TAI groups were matched at a ratio of 1:1 according to age.

^b^Testing for basal FSH, LH and E2 was performed between day 2 and day 4 of the menstrual cycle.

Group characteristics regarding COS and fertilization procedures are shown in **Table 2**. There were no significant differences between the two groups in the protocols and gonadotropin dose of COS, hormone levels on the day of HCG trigger, endometrial thickness, fertilization procedures, timing of embryo transfer, the number of good-quality embryos or embryos transferred. In addition, no significant difference was found in the number of oocytes retrieved (median [interquartile range]: 10 (6–12) *vs.* 11 (7–13), P = 0.091); however, after adjusting for AMH levels, basal FSH levels, basal E2 levels, FT4 levels, TSH levels, and antral follicle count, the number of oocytes retrieved was significantly lower in patients with TAI than in controls (Regression Coefficient, -0.48; 95%CI, -0.06 to -0.90; P=0.025) (**Table 3**).

**Table 2:** Protocols of controlled ovarian stimulation and data of *in vitro* fertilization and embryo transfer.

Characteristics	TAI group (N=778)	Control group (N=778)	P values
Protocol of controlled ovarian hyperstimulation			
Ultralong GnRH agonist	132 (17.0)	110 (14.1)	0.286
Long GnRH agonist	258 (33.2)	278 (35.7)	
Short GnRH agonist	6 (0.8)	3 (0.4)	
GnRH antagonist	382 (49.1)	387 (49.7)	
Gonadotropin dose, mean (SD)	2700 (2025-3450)	2700 (2025-3525)	0.664
LH on HCG trigger day, median (IQR), mIU/ml	1.0 (0.6-2.1)	1.0 (0.6-2.1)	0.878
E2 on HCG trigger day, median (IQR), mIU/ml	6050.0 (4172.0-9216.0)	5998.0 (4240.0-8682.0)	0.668
P on HCG trigger day, median (IQR), mIU/ml	2.0 (1.3-2.7)	2.0 (1.4-2.7)	0.990
Endometrial thickness, mean (SD), mm	11 (10-12)	11 (10-12)	0.893
No. of retrieved oocytes per cycle, mean (SD)	10 (7-13)	11 (8-14)	0.091
Fertilization, No (%)			
IVF	485 (62.3)	492 (63.2)	0.714
ICSI	293 (37.7)	286 (36.8)	
No. of good-quality embryos per cycle, median (IQR)^a^	4 (2-6)	4 (2-6)	0.988
No. of embryos transferred, No. (%)			
1	122 (15.7)	110 (14.1)	0.393
2	656 (84.3)	668 (85.9)	
Timing of embryo transfer, No. (%)			
Day 3	720 (92.5)	711 (91.4)	0.401
Day 5	58 (7.5)	67 (8.6)	

LH, luteinizing hormone; E_2_, estradiol; P, progesterone; IQR, interquartile range; SD, standard deviation.

a Embryos were evaluated on the third day after fertilization. Good-quality embryos were all developed from 2 pronuclei zygotes and met the following criteria:

(1) had more than 5 blastomeres; (2) size difference was less than 20%; and (3) fragmentation was less than 50%.

**Table 3:** Linear regression analysis of the number of oocytes retrieved in patients with TAI compared with controls.

	Regression Coefficient (95%CI)	P Values
TAI *vs.* Control	-0.48 (-0.06 to -0.90)	0.025
FT4	-0.53 (-1.86 to 0.79)	0.430
TSH	-0.01 (-0.17 to 0.16)	0.922
Basal FSH	-0.28 (-0.38 to -0.19)	<0.001
Basal E_2_	-0.01 (-0.008 to -0.001)	0.007
AMH	0.47 (0.36 to 0.59)	<0.001
Antral follicle count	0.19 (0.13 to 0.24)	<0.001

AMH, Anti-Mullerian hormone; FT4, free thyroxine; TSH, thyroid-stimulating hormone; FSH, follicle stimulating hormone; E_2_, estradiol; TAI, thyroid autoimmunity.

There were no significant differences between the TAI and control groups in the rates of clinical pregnancy (44.2% *vs.* 45.1%, P = 0.721), miscarriage (14.5% *vs.* 14.2%, P = 0.916), live birth (37.8% *vs.* 38.7%, P = 0.715), or preterm delivery (16.0% *vs.* 15.6%, P = 0.901), even after adjusting for FT4 and TSH levels, as well as the types of thyroid antibodies (**Tables 4** and **5**). Birth weight in singleton pregnancy was not significantly different between the two groups; however, birth weight in twin pregnancy was significantly lower in the TAI group than in the control group (median [interquartile range]: 2500 [2200–2760] vs. 2650 [2300–2900], P = 0.003) (**Table 4**).

**Table 4:** Pregnancy outcomes in the TAI and control groups.

Outcomes	TAI group (N=778)	Control group (N=778)	P values
Clinical pregnancy^a^, No. (%)	344/778 (44.2)	351/778 (45.1)	0.721
Miscarriage^b^, No. (%)	50/344 (14.5)	50/351 (14.2)	0.913
Live birth^c^, No. (%)	294/778 (37.8)	301/778 (38.7)	0.715
Preterm delivery^d^, No. (%)	47/294 (16.0)	47/301 (15.6)	0.901
Singleton	11/47 (5.3)	9/47 (4.2)	0.616
Twin	36/47 (41.9)	38/47 (42.7)	0.911
Birth weight-g			
Singleton	3385 (3100-3600)	3400 (3120-3650)	0536
Twin	2500 (2200-2760)	2650 (2300-2900)	0.003

^a^Clinical pregnancy was defined as at least one gestational sac in the uterus at 35 days after embryo transfer, as identified on ultrasonography.

^b^Miscarriage was defined as loss of clinical pregnancy before 28 weeks gestation.

^c^Live birth was defined as the delivery of at least one survived newborn, irrespective of gestation duration.

^d^Preterm delivery was defined as delivery of a living fetus before 37 weeks.

**Table 5:** Logistic regression analysis of factors associated with pregnancy outcomes.

	Clinical pregnancy		Miscarriage		Live birth		Preterm delivery	
	OR (95%CI)	P Values	OR (95%CI)	P Values	OR (95%CI)	P Values	OR (95%CI)	P Values
FT4	0.92 (0.49-1.75)	0.807	0.32 (0.08-1.33)	0.119	1.20 (0.62-2.30)	0.589	0.65 (0.16-2.61)	0.541
TSH								
<2.5	NA	NA	NA	NA	NA	NA	NA	NA
≥2.5	1.27 (1.03-1.58)	0.029	1.06 (0.68-1.65)	0.793	1.22 (0.98-1.52)	0.072	0.92 (0.57-1.47)	0.712
Thyroid antibody								
TPOAb+TGAb+	0.89 (0.70-1.15)	0.381	1.05 (0.62-1.79)	0.850	0.89 (0.69-1.15)	0.380	1.06 (0.61-1.85)	0.842
TPOAb-TGAb+	1.03 (0.77-1.38)	0.827	1.00 (0.55-1.85)	0.991	1.02 (0.76-1.38)	0.877	0.77 (0.39-1.52)	0.450
TPOAb+TGAb-	0.96 (0.68-1.35)	0.818	0.99 (0.48-2.06)	0.978	0.96 (0.68-1.36)	0.822	1.46 (0.73-2.90)	0.286
TPOAb-TGAb-	NA	NA	NA	NA	NA	NA	NA	NA

NA, not applicable.

Based on the types of thyroid antibodies, the TAI group was divided into three subgroups: co-positive for TGAb and TPOAb, isolated positive for TGAb, and isolated positive for TPOAb. Subgroup analysis did not find any significant difference among these three groups and the control group in the rates of clinical pregnancy, miscarriage, live birth, and preterm delivery or live birth weight in singleton pregnancy (**Table 6**). The live birth weight in twin pregnancy was significantly higher in the control group than in each of the three subgroups in the TAI group; however, there was no significant difference among the three subgroups of the TAI group in this regard (**Table 6**).

**Table 6:** Subgroup analysis for pregnancy outcomes according to TGAb/TPOAb.

Outcomes	TGAb+ TPOAb+ (N=371)	TGAb+ TPOAb- (N=244)	TGAb- TPOAb+ (N=163)	TGAb- TPOAb- (N=778)	P values
Clinical pregnancy^a^, No. (%)	160/371 (43.1)	112/244 (45.9)	72/163 (44.2)	351/778 (45.1)	0.900
Miscarriage^b^, No. (%)	24/160 (15.0)	16/112 (14.3)	10/72 (13.9)	50/351 (14.2)	0.995
Live birth^c^, No. (%)	136/371 (36.7)	96/244 (39.3)	62/163 (38.0)	301/778 (38.7)	0.899
Preterm delivery^d^, No. (%)	22/136 (16.2)	12/96 (12.5)	13/62 (21.0)	47/301 (15.6)	0.562
Birth weight-g					
Singleton	3400 (3100-3600)	3400 (302.5-3650)	3300 (3000-3500)	3400 (3115-3650)	0.343
Twin	2500 (2250-2800)	2425 (2160-2675)	2500 (2052.5-2715)	2650 (2300-2900)	0.013

TGAb, thyroglobulin antibody; TPOAb, thyroid peroxidase antibody.

^a^Clinical pregnancy was defined as at least one gestational sac in the uterus at 35 days after embryo transfer, as identified on ultrasonography.

^b^Miscarriage was defined as loss of clinical pregnancy before 28 weeks gestation.

^c^Live birth was defined as the delivery of at least one survived newborn, irrespective of gestation duration.

^d^Preterm delivery was defined as the delivery of a living fetus before 37 weeks.

Based on TGAb titers, patients with isolated positive for TGAb were divided into the high (TGAb ≥ 133.2 IU/ml) and low (TGAb < 133.2 IU/ml) titer groups. There were no significant differences between the two groups in the rates of clinical pregnancy, miscarriage, live birth, and preterm delivery or birth weight in singleton or twin pregnancy (**Table 7**). Based on TPOAb titers, patients with isolated positive for TPOAb were divided into the high (TPOAb ≥ 165) and low (TPOAb < 165) titer groups. No significant difference was observed between the two groups in the rates of clinical pregnancy, miscarriage, live birth, and preterm delivery or birth weight in singleton or twin pregnancy (**Table 8**).

**Table 7:** Subgroup analysis for pregnancy outcomes according to isolated TGAb tertiles.

Outcomes	Low titers	High titers	P Values
Clinical pregnancy^a^, No. (%)	53/122 (43.4)	59/122 (48.4)	0.441
Miscarriage^b^, No. (%)	10/53 (18.9)	6/59 (10.2)	0.189
Live birth^c^, No. (%)	43/122 (35.2)	53/122 (43.4)	0.190
Preterm delivery^d^, No. (%)	5/43 (11.6)	7/53 (13.2)	0.816
Birth weight-g			
Singleton	3500 (2980-3900)	3400 (3065-3565)	0.434
Twin	2495 (2307.5-2750)	2400 (2072.5-2675)	0.274

^a^Clinical pregnancy was defined as at least one gestational sac in the uterus at 35 days after embryo transfer, as identified on ultrasonography.

^b^Miscarriage was defined as loss of clinical pregnancy before 28 weeks gestation.

^c^Live birth was defined as the delivery of at least one survived newborn, irrespective of gestation duration.

^d^Preterm delivery was defined as delivery of a living fetus before 37 weeks.

**Table 8:** Subgroup analysis for pregnancy outcomes according to isolated TPOAb tertiles.

Outcomes	Low titers	High titers	P Values
Clinical pregnancy^a^, No. (%)	38/82 (46.3)	34/81 (42.0)	0.575
Miscarriage^b^, No. (%)	7/38 (18.4)	3/34 (8.8)	0.240
Live birth^c^, No. (%)	31/82 (37.8)	31/81 (38.3)	0.951
Preterm delivery^d^, No. (%)	7/31 (22.6)	6/31(19.4)	0.755
Birth weight-g			
Singleton	3200 (2800-3500)	3300 (3100-3500)	0.356
Twin	2500 (1387.5-2765)	2425 (2052.5-2582)	0.678

^a^Clinical pregnancy was defined as at least one gestational sac in the uterus at 35 days after embryo transfer as identified on ultrasonography.

^b^Miscarriage was defined as loss of clinical pregnancy before 28 weeks gestation.

^c^Live birth was defined as the delivery of at least one survived newborn, irrespective of gestation duration.

^d^Preterm delivery was defined as delivery of a living fetus before 37 weeks.

## Discussion

The thyroid gland plays a critical role in regulating reproductive function and maintaining pregnancy balance. The mechanisms underlying the impact of TAI on IVF/ICSI outcomes from follicular growth to pregnancy remain unclear, but currently, there are two hypotheses. First, the presence of TAI triggers a subtle deficiency of thyroid reserve and consequently, leading to a reduced capacity of the thyroid gland to adapt to the augmented demand in the process of COS and early pregnancy. Second, TAI is an immune disorder, and thyroid antibodies with different titers indicates an immune imbalance that impairs the establishment of immune tolerance during pregnancy.

We compared ovarian reserve between the two groups based on the AMH level, antral follicle count, basal FSH levels and basal E2 levels. The AMH and E2 levels were significantly higher in patients with TAI than in age-matched controls, indicating a higher ovarian reserve in patients with TAI. Although a higher ovarian reserve was observed in patients with TAI, the number of oocytes retrieved was significantly lower in patients with TAI than in controls after adjusting for the levels of AMH, basal FSH, basal E2 and FT4, and TSH and antral follicle count. Immune imbalance and abnormal thyroid function during COS may affect follicle development in patients with TAI. In a recent study, Monteleone et al. revealed the expression of thyroid peroxidase in human granulosa cells by immunocytochemistry (14). Moreover, they collected the follicle fluid in women after oocyte retrieval and detected the presence of TPOAb and TGAb in the follicle fluid in women with TAI. The presence of thyroid antibodies in the follicular fluid suggests that antibody-mediated cytotoxicity impairs oocytes (6).

Several studies have reported the presence of TH and TSH in the human follicular fluid, with similar levels in the serum (7, 8). TH may affect follicle development by promoting granulosa cell proliferation and triggering some alterations in the expression of genes involved in steroidogenesis (9–12). The change in thyroid function during COS has been widely investigated, and the elevated trend of TSH level has been reported in many studies, although the results for FT4 are inconsistent (13, 15). A prospective study conducted by Poppe et al. investigated different thyroid functions in women with or without TAI during COS and demonstrated a significant difference in TSH levels between the two groups (16). Different TH levels between women with and without TAI may provide a possible explanation for the decreased number of oocytes retrieved in patients with TAI. In our study, a significant difference was observed in FT4 and TSH levels between the two groups before COS, suggesting that the thyroid plays a possible role in affecting follicle development in the TAI group. However, we could not detect the difference in thyroid function between the two groups during COS due to the study’s retrospective design. Therefore, a further prospective study may be required to determine whether different levels of thyroid function affect follicle development and decrease the number of oocytes retrieved.

The association between TAI and IVF/ICSI outcomes has been reported in several studies, but the results remain controversial. Two studies revealed that the clinical pregnancy rate was significantly reduced in women with TAI (17, 18), whereas five studies failed to show this difference (19–23). Three studies reported that the presence of TAI decreased the birth or delivery rate (17, 18, 22), but five studies did not detect this effect (19, 20, 24–26). Two studies revealed that the miscarriage rate was increased in women with TAI (21, 22), but eight studies reported no difference in the miscarriage rate between women with and without TAI (17, 19, 20, 23–27). In our study, we found no significant differences between the two groups in the rates of clinical pregnancy, miscarriage, preterm delivery, or live birth, even after adjusting for FT4 levels, TSH levels, and thyroid antibody types. Subgroup analysis also failed to detect differences in pregnancy outcomes among patients with different types or titers of thyroid antibodies.

Although the birth weight in singleton pregnancy was not different between the TAI and control groups in our study, the birth weight in twin pregnancy was significantly lower in patients with TAI than in controls. Decreased birth weight might result from abnormal thyroid function during the pregnancy period. Adequate TH availability during pregnancy is crucial for fetal growth and development. Fetal demand for TH during early pregnancy predominantly depends on the placental transfer of maternal TH because the fetal thyroid gland is non-functional until 18–20 weeks of pregnancy. Elevated production of thyroid-binding globulin and expression of deiodinase 3 in the placenta further increase the demand for TH availability. HCG, produced specifically in early pregnancy, can stimulate the thyroid gland *via* binding to the TSH receptor, thus triggering an elevation in serum FT4 levels and a subsequent feedback suppression of TSH secretion. The HCG-mediated thyroid response is important for maintaining adequate TH during early pregnancy (28). A recent study reported that compared with singleton pregnancy, twin pregnancy was associated with a lower TSH levels and a higher FT4 levels during early pregnancy, which were due to increased stimulation of the thyroid gland because of higher HCG levels (29). Thus, a stronger thyroidal response to HCG is needed in twin pregnancy to maintain higher TH. However, the presence of thyroid antibodies has been proved to be a risk factor associated with impairment of the thyroid response to HCG. Notably, an association between HCG and FT4 or TSH was impaired in women with positive thyroid antibodies, compared with in those with negative thyroid antibody (5, 30). In addition, Zhang et al. stated that impaired thyroid response to HCG in the first trimester led to fetal growth restriction and lower crown-rump length (31). Thus, an impaired thyroidal response might lead to lower weight in twin pregnancy in our study.

There are some limitations in our study. First, our study was a retrospective study; thus, the presence of biases involved in retrospective data collection cannot be excluded. Second, this study only included the participants seeking IVF/ICSI treatment due to tubal or male factors; hence, our study can only explain the association of TAI and pregnancy outcomes in patients without other reproductive disorders, and the impact of TAI on IVF/ICSI outcomes in patients with combined reproductive and endocrine diseases needs to be further investigated. Lastly, our study did not investigate the use of levothyroxine because of the retrospective design; however, our previous study has shown that the use of levothyroxine did not affect IVF/ICSI outcomes (32).

In summary, the study revealed that the presence of TAI did not affect embryo quality and pregnancy outcomes but decreased the number of oocytes received and birth weight in twin pregnancy. There was no association between the types and titers of thyroid antibodies and adverse IVF/ICSI outcomes. Further prospective studies are needed to illustrate the mechanisms underlying the impact of TAI on follicle development and fetal growth.

## Data Availability Statement

The raw data supporting the conclusions of this article will be made available by the authors, without undue reservation.

## Ethics Statement

The studies involving human participants were reviewed and approved by Peking University Third Hospital Medical Science Research Ethics Committee. The ethics committee waived the requirement of written informed consent for participation. Written informed consent was obtained from the individual(s) for the publication of any potentially identifiable images or data included in this article.

## Author Contributions

NH, JQ, and HC developed the conception of the study and all authors contributed to the research discussion. NH, LC, YL, HW, and RL took part in patients follow-up and contributed to the data analysis. NH wrote the initial draft of the paper and all authors contributed to manuscript revision. All authors contributed to the article and approved the submitted version.

## Funding

This work was supported by the National Natural Science Foundation of China (Grant no. 81871212) and Science Foundation of Peking University Third Hospital (BYSYLXHG2019003).

## Conflict of Interest

The authors declare that the research was conducted in the absence of any commercial or financial relationships that could be construed as a potential conflict of interest.
